# Towards Secure Embodied Communication Management in AI Era: Reputation-Guided Agent Message Exchange

**DOI:** 10.3390/s26092853

**Published:** 2026-05-02

**Authors:** Jiangtao Mu, Li Wan, Zehui Dong, Yong Wei, Zhiwei Xu

**Affiliations:** 1 School of Economics and Management, University of Science and Technology Beijing, Beijing 100083, China; 2 State Grid Beijing Electric Power Supply Company, Beijing 100031, China; 3 Haihe Lab of ITAI, Tianjin 300459, China; 4 Institute of Computing Technology, Chinese Academy of Sciences, Beijing 100190, China; 5 State Grid Information Telecommunication Group Co., Ltd., Beijing 102211, China

**Keywords:** embedded intelligent devices, secure communication management, Man-in-the-Middle Attack, reputation-guided path selection, byzantine resilience

## Abstract

For large-scale embedded sensor-actuator networks, such as robotic swarms deployed over vast areas and other embedded intelligent devices, end-to-end message exchange is often impossible due to their limited communication range, power constraints, and device mobility. Devices, thus, rely on multi-hop relaying, exposing them to Man-in-the-Middle (MitM) attacks where compromised relays tamper with, forge, or inject false messages. The existing countermeasures, including end-to-end encryption or Byzantine consensus, involve high overhead while requiring global coordination and, thus, renders them impractical for time-sensitive message exchange in embedded intelligence. Security management on communication among embodied devices is highly desired. To address this challenge, we propose Reputation-Guided Dynamic Relay Selection (RDRS), a lightweight, distributed countermeasure against MitM attacks that leverages interactive feedback to evaluate reputation of embedded devices. Specifically, each device maintains reputation scores updated via recent interaction success rates with decay factors to counter dynamic adversaries. During exchanging messages, embedded devices select next-hop neighbors weighted by reputation scores, effectively bypassing malicious devices without explicit detection or in-path verification. Comprehensive simulations in embedded sensor-actuator networks demonstrate that RDRS reduces tampering success rate (TSR) by 80–95% compared to the baselines, martians request satisfaction rate (RSR) above 79% even at 40% malicious nodes, and achieves lower delay 64% with comparable overhead.

## 1. Introduction

The fourth industrial revolution, known as Industry 4.0, has transformed manufacturing and industrial operations through the integration of cyber-physical systems and Artificial Intelligence (AI) [1]. Large-scale sensor-actuator networks, such as robotic swarms, drone fleets, unmanned vehicle clusters and other heterogeneous sensor-actuator networks, have taken as key enablers for intelligent industrial applications, and used in environmental monitoring, precision agriculture, and collaborative exploration in hazardous environments [2]. These applications consist of numerous time-sensitive devices equipped with sensing, actuation, and real-time processing capabilities, that are often physically distributed over vast geographic areas. Due to limited communication range, strict latency requirements, and dynamic mobility, such devices must rely on multi-hop message relaying, where intermediate devices forward messages, states, or sensory data on behalf of others, forming ad-hoc communication paths similar to those in wireless sensor networks. This multi-hop message exchange paradigm enhances scalability, yet introduces security vulnerabilities [3]. In particular, any compromised intermediate device can launch Man-in-the-Middle (MitM) attacks by tampering with, forging, or injecting false messages during relaying. Although existing countermeasures—such as end-to-end encryption and Byzantine consensus protocols—have been proposed, they impose prohibitive computational and communication overhead, require global coordination or synchronized updates, and are fundamentally impractical for highly dynamic, asynchronous, and latency-sensitive embedded environments. It is highly desired to study secure communication management on embodied devices. If an intermediate device is compromised—either through physical captured, it can launch Man-in-the-Middle Attack(MitM) during message exchange. Such attacks include tampering with message content, injecting false data, selectively dropping packets, or replaying outdated information, leading to degraded system performance, incorrect collective decisions, or even physical safety risks (e.g., collision in drone swarms [4,5]). These threats [6] are exacerbated in advanced sensor-actuator networks by the time-sensitive nature of embedded devices, high mobility and lack of transparent coordination [7,8].

Unfortunately, existing host-centric security mechanisms, such as end-to-end encryption or global authentication, is insufficient or overwhelming expensive. These approaches primarily fall into two categories: cryptographic defenses and resilient consensus algorithms. Cryptographic methods [9], including digital signatures and proxy-based schemes using lightweight cryptography (e.g., hyperelliptic curve-based proxy signatures for time-sensitive environments), ensure end-to-end integrity but fail to prevent in-path tampering by malicious relays [10,11,12], as intermediates can still modify or forge data before message exchange. Resilient consensus protocols [13], such as Mean-Subsequence-Reduced (MSR) algorithms or Byzantine fault-tolerant variants, allow honest devices to filter out anomalous values from neighbors, achieving approximate agreement even in the presence of Byzantine adversaries. However, these often require synchronous updates, high communication overhead, or assumptions of bounded malicious devices (e.g., fewer than one-third malicious devices). Therefore, they are impractical for highly dynamic, asynchronous embedded systems with strict timing constraints. When devices exchange task-specific messages (e.g., position updates, sensor readings, or coordination commands) over unreliable multi-hop paths, malicious relays can persistently poison messages without being easily detected. Reputation-based mechanisms in related fields, such as wireless sensor networks [14], have shown promise in dynamically avoiding untrustworthy devices through local trust evaluations [15,16], but rarely address the unique challenges of time-sensitive embedded devices. Inspired by existing reputation-based mechanisms, we propose a reputation-guided embodied agent message exchange.

**Figure 1:**
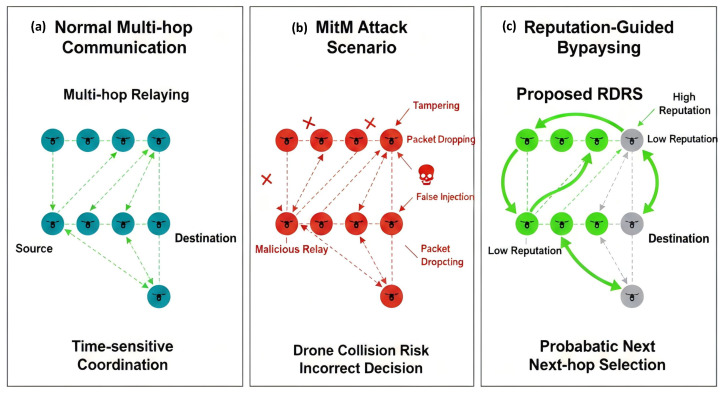
Multi-hop message exchange, Man-in-the-Middle attacks against embodied devices and reputation-based solution: (**a**) Honest relaying forming reliable paths, (**b**) Man-in-the-Middle (MitM) attack where malicious relays tamper with, dropout, or inject false messages, leading to risks (e.g., drone collision), (**c**) Reputation-guided path selection for dynamically bypassing malicious devices.

To address the identified issues, we design a lightweight, distributed Reputation-Guided Dynamic Relay Selection (RDRS) framework that enables secure multi-hop message exchange in time-sensitive embedded sensor-actuator networks, through bypassing malicious relays using local interactive feedback. More specifically, an interaction-driven reputation maintenance protocol is proposed to rapidly adapt to dynamic adversaries, achieving secure embodied communication management in AI era, which is our primary objective to design RDRS. The main contributions of this paper are as follows:

- This paper proposes RDRS, a novel distributed framework that leverages direct feedback from device interactions to achieve secure message relaying in sensor-actuator networks.
- We design interaction-driven reputation updates, where recipients provide explicit or implicit feedback on received message integrity, and senders and intermediates use it to update local reputation scores of one-hop neighbors. These scores are then used to probabilistically select next-hop relays, naturally bypassing malicious devices without requiring global fault localization or heavy cryptographic overhead.
- By integrating optional reinforcement learning for adaptive path perturbation [17], RDRS draws from moving target defense principles in proxy-based enhancements for critical messages, and achieves high resilience against dynamic adversaries while preserving low latency and energy efficiency, as illustrated in Figure 1.

The remainder of this paper is organized as follows. Section 2 reviews related work on multi-hop communication of embodied devices and the related threats. Section 3 presents the detailed design of the proposed RDRS. Section 4 evaluates performance through simulations and provides security analysis. Finally, Section 5 concludes the paper.

## 2. Related Work

### 2.1. Security Threats and Reputation Evaluation

For security threats in multi-device message interaction in embodied devices, existing protection measures mainly focus on two categories: encryption authentication and path optimization. Encryption authentication technology ensures the confidentiality and integrity of messages through cryptographic methods, but it cannot defend against malicious relay devices’ forwarding tampering or denial-of-service attacks [3]. Path optimization technology improves communication reliability through topology reconstruction, redundant path construction, etc. For example, the distributed routing protocol in satellite communication systems can avoid faulty devices through dynamic path adjustment [18,19]. However, these approaches fundamentally operate in network layer and lack the ability to model and evaluate the trustworthiness of intermediate devices. As a result, they cannot explicitly distinguish malicious relays from unreliable but benign nodes, which limits their effectiveness in adversarial environments.

Reputation mechanisms, as an effective tool for identifying malicious behavior and guiding trust-based collaboration, have been widely applied in device conflict resolution and time-critical task allocation scenarios in time-sensitive embodied environments [20,21]. Existing research has constructed reputation evaluation models and combined them with devices’ historical interaction behavior to quantify trust levels, thereby isolating and constraining untrustworthy devices [22]. For example, in human–machine collaboration systems, reputation fusion models based on reinforcement learning can dynamically adjust device interaction strategies to improve collaboration security. Despite their effectiveness in global trust assessment, existing reputation models are typically device-centric rather than path-aware. They evaluate the overall behavior of individual devices but do not capture the fine-grained trust dynamics along multi-hop message transmission paths. Consequently, they cannot directly support secure path selection in scenarios involving malicious relays.

Existing methods either focus on secure communication primitives (encryption, routing) or global trust evaluation, but lack a unified mechanism that jointly considers message transmission paths and dynamic trust propagation. This gap motivates our work on path-aware trust modeling for secure message interaction.

### 2.2. MitM Defenses in Embedded Environment

Man-in-the-Middle attacks are typical security threat in multi-device message interaction in embodied devices, posing a serious challenge to the security of scenarios such as NFC payments and connected vehicles [23,24,25]. Existing research mainly revolve Man-in-the-Middle attacks around three technical paths: distance binding, environmental perception, and physical layer feature verification.

Distance binding is a traditional core technology for defending against malicious relaying. It estimates the physical distance between communicating entities by measuring the round-trip time (RTT) or time-of-flight (ToF) of the signal, thereby identifying relay attacks that exceed the legal range [26,27,28]. The basic distance binding protocol proposed by Brands et al. achieves distance verification through the timing constraints of the challenge-response mechanism. Hancke et al. further adapted it to RFID systems, forming the HKP protocol [29,30]. To improve practicality, subsequent studies introduced error correction codes to handle transmission errors and used an empty challenge mechanism to deal with the random guessing behavior of attackers. However, traditional distance binding faces hardware dependence and accuracy bottlenecks: although ultra-wideband (UWB) technology can improve the accuracy of ToF measurement, it requires dedicated hardware and has a low data transmission rate [31]. In addition, this technology cannot defend against short-range relay scenarios and has limited resistance to advanced attacks such as Mafia fraud [32].

Using built-in environmental sensors to collect scene features and verifying the consistency of the environment between the communicating parties to detect relay attacks has become a research hotspot in recent years [33,34]. Existing solutions mainly use a combination of single or multiple sensors, including GPS positioning [35], sound and light [33], accelerometers [34], temperature [36], etc. For example, Halevi et al. achieved proximity verification by comparing the ambient sound and light data of the communicating parties, and Mehrnezhad et al. proposed a “double-tap” interaction mechanism based on accelerometers, requiring users to tap the terminal continuously to confirm physical proximity [33,34]. These approaches improve performance by leveraging commodity sensors, but they are inherently sensitive to environmental noise and context. Moreover, attackers may spoof or partially replicate environmental signals, reducing robustness in adversarial settings.

The uniqueness and reciprocity of physical layer features based on wireless channels provide a new path for malicious relay detection. This type of method utilizes physical features such as channel state information (CSI) and channel frequency response (CFR) to verify whether the communicating parties are directly interacting, thus, defending against relay attacks at the source [25,34]. Zenger et al. detected decode-forward (DF) relay attacks by quantizing the reciprocity of received signal strength (RSS), and Abubaker et al. further proposed a channel fingerprinting scheme based on OFDM systems, which identifies DF attacks by comparing the correlation of CFR [34,37]. Although physical-layer methods offer strong security guarantees, they rely on strict assumptions about channel reciprocity and require stable wireless conditions. Their performance may degrade in dynamic or noisy environments, which are common in embodied systems. To better illustrate the limitations of existing MitM defenses, we summarize representative approaches in Table 1.

## 3. Reputation-Guided Dynamic Relay Selection

**Figure 2:**
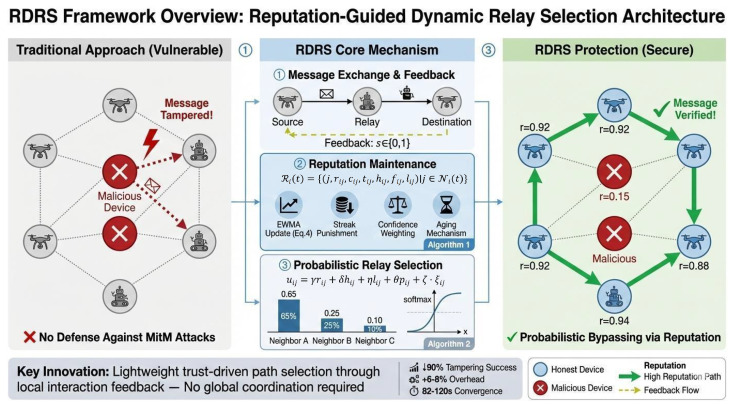
Overall architecture of RDRS framework.

This section presents the proposed Reputation-Guided Dynamic Relay Selection (RDRS) framework, which enables secure and reliable multi-hop communication in large-scale, time-sensitive networks of embedded intelligent devices. By modeling intermediate devices as dynamic relays, RDRS effectively mitigates MitM attacks while incurring minimal latency, computational, and communication overheads. The design leverages direct interactive feedback between devices to build and maintain trust, adapting reputation-based relay bypassing to identifier-less, highly dynamic embedded intelligent device topologies, as illustrated in Figure 2. Figure 2 provides a comprehensive overview of the RDRS architecture. The left panel illustrates the vulnerability of traditional multi-hop relaying to MitM attacks, the center panel details the core feedback-driven reputation maintenance and probabilistic selection mechanisms, and the right panel demonstrates secure message delivery via reputation-guided bypassing of malicious relays. Additional enhancements integrate lightweight hyperelliptic curve cryptography (HCC)-based proxy signatures for critical messages [38] and reinforcement learning-driven moving target defense for controlled path randomization [39].

### 3.1. Design Objectives, Principles, and Threat Model

#### 3.1.1. Design Objectives

The Reputation-Guided Dynamic Relay Selection (RDRS) framework is designed to meet the stringent requirements of large-scale time-sensitive embedded systems. It achieves minimal overhead through lightweight operations: reputation tables are limited to $O(|N_{i}|)$ entries (typically $|N_{i}|\;<20$), updates use constant-time O(1) exponentially weighted moving averages (EWMA) with asymmetric punishment, and next-hop selection employs probabilistic softmax with $O(|N_{i}|)$ complexity. By avoiding per-hop cryptographic verification and relying on asynchronous feedback from device interactions, RDRS remains feasible on latency-sensitive platforms such as drone swarms. The framework is fully distributed, requiring no centralized authorities, global topology knowledge, or synchronized clocks; each device autonomously maintains reputation based solely on one-hop interactions and recipient feedback. Rapid adaptation is enabled by a hybrid reputation model that aggressively degrades scores for malicious behavior via streak-based exponential punishment multipliers ($\beta^{f}$) and confidence weighting, while permitting gradual recovery of benign or transiently faulty devices through periodic aging toward a neutral value.

RDRS further ensures resilience under partial compromise (malicious relays <30%) by probabilistically routing along high-reputation paths, bounding poisoning success rates even under static or combined attacks. Extensibility supports integration of lightweight cryptographic primitives (e.g., delegated hyperelliptic curve proxy signatures) and learning-based adaptations (e.g., Q-learning or WoLF-PHC for tuning temperature γ and punishment β). Mobility tolerance is achieved through hysteresis reward for recent successful relays, EWMA-smoothed link quality estimates, and temporary reverse-path caching for feedback delivery. At its core, RDRS follows the principle of probabilistic reputation-guided bypassing: devices dynamically evaluate neighbor trustworthiness from local observations and interaction feedback, preferentially forwarding messages along reliable paths to naturally isolate malicious relays without explicit detection or costly per-hop checks.

#### 3.1.2. Principles

The core principles of the Reputation-Guided Dynamic Relay Selection (RDRS) framework center on an interaction-oriented defense paradigm, where the primary goal is to dynamically exclude malicious relays from forwarding paths through feedback gathered directly from device-to-device interactions, rather than relying on computationally expensive in-network verification at every hop. In time-sensitive embedded intelligent devices, this translates to treating intermediate devices as dynamic relays that can be probabilistically bypassed based on locally maintained reputation scores derived from recipient feedback, thereby ensuring secure multi-hop message propagation without requiring global identifiers, centralized coordination, or per-hop cryptographic checks.

RDRS leverages asynchronous feedback from message recipients—who perform lightweight local verification using contextual plausibility checks (e.g., consistency with known task semantics or sensor redundancy), sequence numbering, or optional hyperelliptic curve-based proxy signatures delegated to trusted high-capability anchors [38]—to drive reputation updates at senders and intermediate relays. This interaction-driven approach, combined with a hybrid reputation model featuring asymmetric exponentially weighted moving averages (EWMA), streak-based exponential punishment multipliers, confidence saturation, short-term hysteresis reward for recent successes, and periodic aging toward a neutral default, enables rapid demotion of misbehaving relays while allowing gradual recovery for devices exhibiting transient faults or reformed behavior. Path selection decisions employ a stabilized softmax distribution over current reputation scores, augmented with optional link quality estimates and controlled exploration, ensuring that traffic naturally converges toward trustworthy paths even in highly mobile topologies. By incorporating insights from game-theoretic moving target defense in vehicular networks [39], RDRS further supports optional reinforcement learning adaptations (e.g., Q-learning or WoLF-PHC) for dynamic parameter tuning and path randomization, providing a lightweight yet adaptive foundation that balances security, efficiency, and robustness across diverse embedded deployment scenarios.

#### 3.1.3. Threat Model

We adopt a realistic threat model in which adversaries can compromise a bounded fraction of devices (typically assumed to be less than 30–40% to preserve an honest majority), granting them the ability to launch sophisticated MitM. This threshold is chosen because it ensures reliable convergence of local reputation scores via interactive feedback and aligns with classic Byzantine fault-tolerance bounds. When the malicious nodes remains less than this threshold, RDRS probabilistically isolates compromised relays with high effectiveness. If malicious activity exceeds 40%, the system experiences graceful degradation—higher TSR and lower RSR, yet still outperforms random forwarding, shortest-path, and consensus baselines by favoring residual high-reputation honest paths, as shown in our scalability experiments. Meanwhile, honest devices faithfully perform lightweight local verification and provide accurate feedback, ensuring updates reflect ground-truth reputation.

Compromised devices may actively tamper with or forge relayed messages, altering payloads to inject false sensory data, corrupted coordination commands, or poisoned content, potentially leading to incorrect collective decisions or physical safety hazards in embedded deployments. They can also selectively drop or delay packets, implementing gray-hole or timing-based attacks to disrupt time-sensitive tasks while evading immediate detection. Furthermore, adversaries may inject false feedback, performing bad-mouthing attacks against honest relays or self-promoting their own reputation through sybil-like collusion, aiming to manipulate reputation convergence and force traffic through malicious paths. Finally, attackers are permitted to exhibit dynamic and intermittent behavior, such as alternating between honest and malicious actions, periodically switching control among different compromised devices, or adapting their strategy based on observed network responses—capabilities that mirror real-world advanced persistent threats. Honest devices, in contrast, faithfully perform lightweight local verification upon message reception (leveraging task-specific contextual checks, multi-source redundancy, checksums, or optional lightweight proxy signatures) and generate accurate binary or probabilistic feedback, ensuring that reputation updates reflect ground-truth outcomes. This threat model deliberately excludes scenarios involving total network compromise or unbreakable cryptographic breaches, focusing instead on practical Byzantine faults where the honest majority enables probabilistic isolation of malicious relays through reputation-guided message forwarding, providing strong security guarantees under realistic deployment assumptions.

We consider a threat model where adversaries can compromise a fraction of devices, enabling them to:

- Tamper with or forge relayed messages during device interactions.
- Selectively drop or delay packets.
- Inject false feedback (sybil-like or bad-mouthing attacks).
- Exhibit dynamic behavior (e.g., intermittent malicious or switching compromised devices).

### 3.2. Reputation Maintenance Protocol

**Figure 3:**
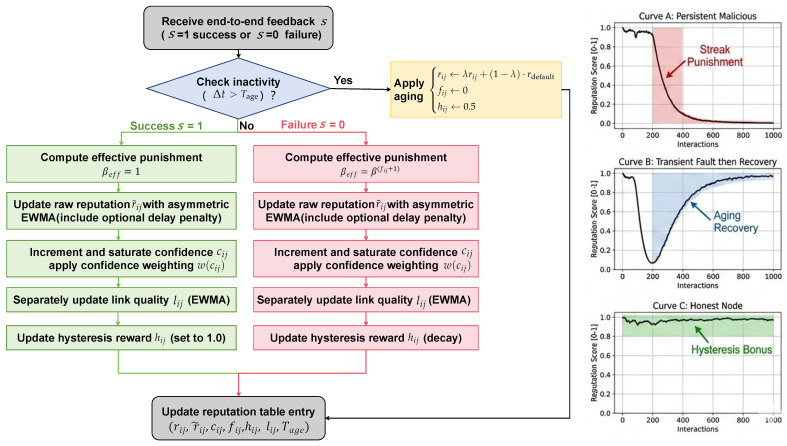
Reputation maintenance and update process in RDRS.

The reputation maintenance protocol in the Reputation-Guided Dynamic Relay Selection (RDRS) framework integrates a sophisticated multi-mechanism approach to ensure robustness, rapid response to malicious behavior, and long-term adaptability in highly dynamic embedded intelligent environments, as shown in Figure 3. This hybrid model combines five complementary components: (1) asymmetric exponentially weighted moving average (EWMA) for smooth baseline tracking of trustworthiness, (2) streak-based escalated punishment to aggressively demote persistent attackers, (3) confidence-weighted saturation to address cold-start vulnerabilities and prevent over-trusting sparsely observed neighbors, (4) periodic aging to facilitate gradual reputation recovery for devices exhibiting transient faults or prolonged inactivity, and (5) short-term hysteresis reward coupled with independent link quality estimation for positive reinforcement of recent reliable performance.

To implement the above components, the framework relies on the following definitions about honest devices and local reputation tables of embodied devices. Honest devices perform lightweight local verification (e.g., contextual plausibility checks, redundancy, or optional signatures) and provide accurate feedback.

Each device *i* maintains a local reputation table:

(1) $$R_{i}\left(t\right)=\left\{\left(j,r_{ij},c_{ij},t_{ij},h_{ij},f_{ij},l_{ij}\right)|j\in N_{i}\left(t\right)\right\},$$

where, each tuple corresponds to one-hop neighbor *j*. The variables are summarized as follows:

- $r_{ij}\in \left[0,1\right]$: reputation score
- $c_{ij}$: confidence level (interaction count)
- $t_{ij}$: last interaction timestamp
- $h_{ij}$: hysteresis reward (recent success memory)
- $f_{ij}$: consecutive failure count
- $l_{ij}$: link quality estimate

Upon receiving end-to-end feedback s∈{0,1} from the message recipient through device interactions (where s=1 indicates successful verification and s=0 denotes detected tampering or failure) for a message forwarding operation involving neighbor *j*, the device first checks for inactivity and applies an aging mechanism to prevent stale reputations from indefinitely influencing message forwarding decisions:

(2) $$\mathrm{if}\;\Delta t=t_{\mathrm{current}}-t_{ij}>T_{\mathrm{age}},\;\left\{\begin{array}{c} r_{ij}\leftarrow \lambda r_{ij}+\left(1-\lambda \right)\cdot r_{\mathrm{default}}\\ f_{ij}\leftarrow 0\\ h_{ij}\leftarrow 0.5 \end{array}\right.$$

where typical values are λ=0.99, $r_{\mathrm{default}}=0.5$, and $T_{\mathrm{age}}=300$ s. This step also resets the failure streak counter $f_{ij}$ and moderately reinitializes the hysteresis reward $h_{ij}$.

Next, the effective punishment multiplier is computed to enable exponential penalty scaling for consecutive malicious events detected via interaction feedback:

(3) $$\beta_{\mathrm{eff}}=\left\{\begin{array}{cc} 1 & \mathrm{if}\;s=1\\ \beta^{\left(f_{ij}+1\right)} & \mathrm{if}\;s=0 \end{array}\right.,\;\beta \in \left[2,4\right]$$

with a recommended base β=3. This streak-based mechanism ensures that isolated incidents incur moderate penalties while persistent tampering detected through device interactions triggers rapid reputation collapse.

The raw reputation score is then updated using an asymmetric EWMA formulation that optionally incorporates soft evidence from timing anomalies:

(4) $${\tilde{r}}_{ij}=\alpha r_{ij}+\left(1-\alpha \right)\left(\beta_{\mathrm{eff}}\cdot s\right)+\kappa \left(1-s\right)\cdot \Delta_{\mathrm{delay}}$$

where α∈[0.85,0.95] controls smoothing, and the delay penalty term $\kappa \left(1-s\right)\cdot \Delta_{\mathrm{delay}}$ (with small κ≈0.01) accounts for selective delay attacks detected through excessive latency relative to historical baselines. Confidence counter $c_{ij}$ is incremented and saturated at $c_{\max}=50$:

(5) $$c_{ij}\leftarrow \min\left(c_{\max},c_{ij}+1\right),\;w\left(c_{ij}\right)=1-\exp\left(-c_{ij}/\tau \right),\;\tau =10$$

The final reputation is confidence-weighted to mitigate premature trust in new or infrequently observed neighbors:

(6) $$r_{ij}\leftarrow w\left(c_{ij}\right)\cdot {\tilde{r}}_{ij}+\left(1-w\left(c_{ij}\right)\right)\cdot r_{\mathrm{default}}$$

Link quality $l_{ij}\in \left[0,1\right]$ (e.g., normalized packet delivery ratio or RSSI) is maintained separately via EWMA to provide an independent reliability signal:

(7) $$l_{ij}\leftarrow \mu l_{ij}+\left(1-\mu \right)\cdot pdr,\;\mu =0.9$$

Hysteresis $h_{ij}$ is set to 1.0 on success and exponentially decayed for all other neighbors each update cycle using decay factor ϕ=0.9.

This comprehensive protocol leverages multi-factor feedback directly from device-to-device interactions, integrating insights from lightweight proxy schemes in time-sensitive environments [38] and adaptive parameter tuning in game-theoretic moving target defense [39], yielding a highly resilient reputation system capable of withstanding sophisticated static, dynamic, and feedback-manipulation attacks while remaining computationally lightweight for time-sensitive embedded devices.The detailed reputation update procedure, incorporating multi-factor integration, is presented in Algorithm 1.
**Algorithm 1** Reputation Update with Multi-Factor Integration
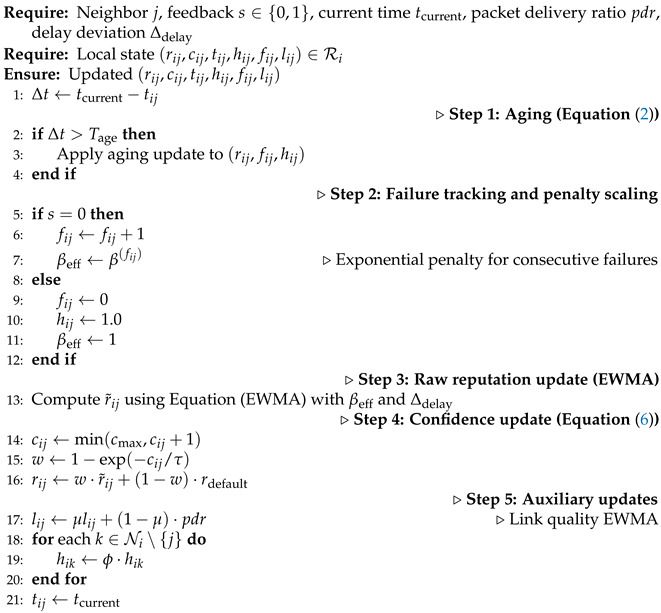


### 3.3. Dynamic Relay Selection and Path Management

The dynamic relay selection and path management components of the Reputation-Guided Dynamic Relay Selection (RDRS) framework are designed to achieve efficient, secure, and adaptive multi-hop message exchange in highly mobile embedded systems by intelligently combining multiple trustworthiness signals derived from device interactions into a unified next-hop decision process. The selection mechanism leverages probabilistic message forwarding based on reputation scores built from direct interaction feedback, prioritizing relays with proven reliability while maintaining controlled exploration to discover potentially better paths or recover from transient reputation degradation.

The utility score for each one-hop neighbor *j* is computed as a weighted linear combination of several complementary factors:

(8) $$u_{ij}=\gamma r_{ij}+\delta h_{ij}+\eta l_{ij}+\theta p_{ij}+\zeta \cdot \xi_{ij}$$

where γ>0 (typically 4–6) emphasizes long-term reputation $r_{ij}$, δ≥0 (typically 1–3) weights the short-term hysteresis reward $h_{ij}$, η≥0 incorporates independent link quality estimate $l_{ij}$ (e.g., EWMA-smoothed packet delivery ratio or normalized RSSI), θ≥0 optionally adds a priority reward $p_{ij}\in \left\{0,1\right\}$ for predefined high-capability anchor devices (analogous to trusted anchors in time-sensitive environments [38]), and $\zeta \cdot \xi_{ij}$ introduces a controlled random exploration term $\xi_{ij}∼\mathrm{Uniform}\left(0,\epsilon_{\mathrm{expl}}\right)$ with small ζ (e.g., $\epsilon_{\mathrm{expl}}=0.1$) to prevent premature convergence to suboptimal paths and support moving target defense principles [39].

To ensure numerical stability and avoid overflow in exponentiation, the softmax probabilities are computed using the log-sum-exp trick.

Path management employs temporary reverse-path entries to enable efficient feedback delivery along the established forward path when possible. When a device forwards an outgoing message, it records the previous hop and a message identifier in a temporary table. Upon receiving corresponding feedback from the recipient (or downstream relay), devices preferentially follow the recorded reverse path if available; otherwise, they independently re-select a next-hop. Verified messages are opportunistically cached in a local buffer with limited size (LRU eviction) to satisfy potential future similar requests from neighbors, reducing latency and communication overhead.The next-hop selection and forwarding process is detailed in Algorithm 2.
**Algorithm 2** Next-Hop Selection and Forwarding**Require:** Message *m*, type ∈{forward,feedback}, priority**Ensure:** Selected next-hop $j^{*}$1:Compute utilities u[j] for all $j\in N_{i}$ using Equation (8)2:**if** priority = high and anchor $a\in N_{i}$ **then**3:      $u\left[a\right]\leftarrow u\left[a\right]+\theta_{\mathrm{anchor}}$4:**end if**5:Compute probabilities P[j] via stabilized softmax over u[j]6:Apply exploration floor ϵ and renormalize *P*7:Sample $j^{*}∼\mathrm{Categorical}\left(P\right)$8:**if** type = forward **then**9:      Record reverse-path entry for *m*10:**else**11:      **if** reverse-path exists **then**12:            Follow recorded path13:      **else**14:            Re-sample $j^{*}$15:      **end if**16:**end if**17:Forward *m* to $j^{*}$ and update $h_{ij^{*}}\leftarrow 1.0$18:**return** 
$j^{*}$

This integrated approach ensures low-latency message forwarding along high-trust paths while preserving opportunistic caching benefits and robustness against topology changes in dynamic embedded interactions.

## 4. Experimental Evaluation

In this section, we evaluate the proposed Reputation-Guided Dynamic Relay Selection (RDRS) framework via simulations, and emulate realistic operating conditions of time-sensitive embedded intelligent devices. The evaluation is based on the sim4DistrDL [40] discrete-event simulator, which integrates a deep learning module and a network simulation module to facilitate simulation of DNN-based distributed applications. sim4DistrDL is a NS3-based simulation framework and, thus, can support Device-in-the-Loop testing, facilitating rapid embodied device deployment for classification, regression, and prediction tasks. The simulator is configured with mobile devices featuring dynamic neighbor discovery, latency-aware per-device local message caches (1–5 MB), temporary reverse-path tables with configurable timeouts, and custom message exchange strategies incorporating reputation-guided probabilistic selection. This setup effectively models embedded devices (e.g., embedded drones, ground robots, or heterogeneous sensor-actuator networks) as mobile entities in large-scale, intermittently connected network simulation supporting multi-hop message relaying and opportunistic caching.

### 4.1. Simulation Setup

#### 4.1.1. Network Topology and Mobility Models

To emulate diverse real-world deployment scenarios in large-scale time-sensitive embedded intelligent devices, evaluations are conducted using sim4DistrDL [40] across two complementary topologies. Sim4DistrDL discrete-event simulator, which integrates a deep learning module and a network simulation module to facilitate simulation of DNN-based distributed applications on embodied devices. To enhance realism and practical credibility, sim4DistrDL loads actual pre-trained DNN models (for target search and target recognition tasks) directly from real-world embodied devices such as drones and ground robots, while emulating their hardware characteristics, sensor data, and actuation behaviors. To ensure full methodological transparency and reproducibility, detailed dataset size, annotation procedures, and DNN training hyperparameters are provided in Section 4.1.4.

The grid topology arranges 100 devices in a 10×10 array with 100 m inter-device spacing, modeling stable, structured environments such as warehouse robotics or precision agriculture monitoring. Conversely, the random topology uniformly distributes 100–300 devices within a 2000×2000 m^2^ area and applies the Random Waypoint mobility model (speeds 5–20 m/s, pause times 0–10 s), capturing highly dynamic swarm operations typical of search-and-rescue missions or urban drone fleets. Among these devices, only the device that requests tasks and the one that responses the request are physical devices, while the others are virtual devices simulated by Sim4DistrDL. Wireless communication assumes a fixed 250 m transmission range, IEEE 802.11p-like parameters (6 Mbps nominal rate), and CSMA/CA channel access.

#### 4.1.2. Malicious Behavior Models

Performance is assessed under four increasingly sophisticated attack scenarios inspired by MitM in distributed embedded intelligent devices and vehicular networks [38,39]:

- Static Malicious Relays: 10–40% of intermediate devices always tamper with relayed message content upon receiving a message.
- Dynamic Intermittent Malicious: Compromised devices alternate between honest and malicious forwarding with tampering probability 0.7 over 50-s cycles.
- False Feedback Injection: Malicious devices systematically send negative feedback for correctly forwarded messages (bad-mouthing attack) while optionally self-promoting honest-like feedback for their own tampered forwards.
- Pure Bad-Mouthing Attack: 10–40% malicious devices inject only false negative feedback for messages correctly forwarded by honest relays (systematic reputation manipulation via bad-mouthing), without performing any content tampering, packet dropping, or self-promotion, to explicitly isolate the impact of reputation attacks.
- Combined Adversarial Scenario: Simultaneous deployment of 20% static tamperers and 10% false-feedback injectors to evaluate resilience against multi-vector threats.

#### 4.1.3. Baseline Comparison Methods

RDRS is benchmarked against a comprehensive set of representative approaches:

- Random Message Forwarding (RF): Uniform random next-hop selection among available neighbors.
- Shortest-Path Greedy Geographic Forwarding (SP): Static path selection vulnerable to malicious interception.
- Mean-Subsequence-Reduced (MSR) Consensus [13]: Outlier-filtering approximate agreement with high communication overhead.
- RDRS-NoHyst: Ablated variant of RDRS without hysteresis reward and streak-based punishment to isolate component contributions.

#### 4.1.4. Parameter Configuration

Core RDRS hyperparameters are fixed at α=0.9 (EWMA smoothing), β=3 (base punishment multiplier), γ=5 (reputation exploitation weight), δ=2 (hysteresis weight), η=1 (link quality weight), minimum exploration floor ϵ=0.01, and aging threshold $T_{\mathrm{age}}=300$ s. For reproducibility, the exchanged messages were generated from the public CValues dataset [41], which contains 14,500 Chinese-language samples originally designed for evaluating LLM safety and responsibility. We randomly sampled 2000 query-response pairs that are semantically relevant to embodied coordination tasks. Annotation was performed automatically via rule-based contextual plausibility checks integrated in sim4DistrDL; no manual annotation was required because the dataset provides pre-labeled safety scores.

The pre-trained DNN models loaded in sim4DistrDL for target search and recognition tasks follow standard architectures. These models were originally trained on 5000 real-world labeled images captured from drone/robot platforms (ImageNet pre-training + domain-specific fine-tuning). Training parameters were: learning rate $1\times 10^{-3}$, batch size 32, Adam optimizer ($\beta_{1}=0.9$, $\beta_{2}=0.999$), 50 epochs, and cross-entropy loss. In our simulations, the pre-trained checkpoints were loaded directly without further training to faithfully emulate hardware behavior on embodied devices. All simulation results are reported as averages over 30 independent runs with 95% confidence intervals computed via the Student’s t-distribution.

### 4.2. Performance Metrics

Evaluation focuses on five key metrics that collectively characterize security effectiveness, usability, and efficiency:

- Tampering Success Rate (TSR): Ratio of tampered messages successfully received and accepted by recipients relative to total delivered messages.
- Request Satisfaction Rate (RSR): Percentage of issued requests fulfilled with contextually or cryptographically verified valid responses.
- Average End-to-End Delay: Mean latency from request issuance until reception of valid corresponding response, measured only for satisfied requests.
- Communication Overhead: Total number of transmitted packets per second per device, encompassing requests, responses, feedback messages, and optional authentication overhead.
- Convergence Time: Simulated time required for TSR to stabilize below a 5% threshold following attack initiation, indicating reputation system responsiveness.

The extensive simulation campaign validates RDRS’s superior resilience across static, dynamic, and combined adversarial settings while maintaining overhead comparable to unprotected baselines, confirming its practicality for deployment on time-sensitive embedded platforms.

### 4.3. Results and Analysis

The simulation results provide compelling evidence of the superior performance of the Reputation-Guided Dynamic Relay Selection (RDRS) framework across a wide range of adversarial conditions and operational scenarios, demonstrating its effectiveness in mitigating MitM while preserving low latency and overhead characteristic of lightweight embedded communication systems.

#### 4.3.1. Effectiveness Against Static Malicious Relays

**Figure 4:**
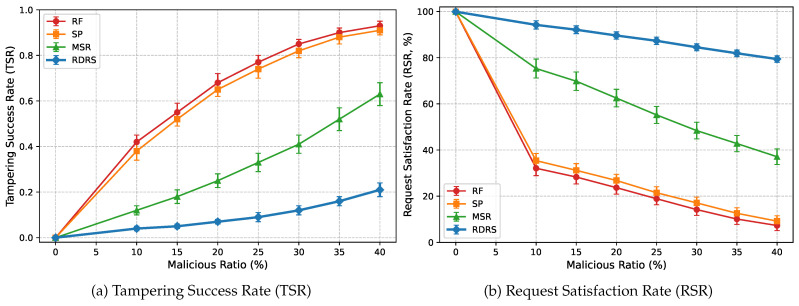
Performance under static malicious relays (Random Topology, 200 devices, averaged over 30 runs). RDRS achieves the lowest TSR (below 0.21 at 40% malicious) and highest RSR (above 79% at 40% malicious), significantly outperforming baselines.

In static attack scenarios, where a fixed percentage of relays consistently tamper with forwarded message content, RDRS exhibits exceptional resilience by rapidly demoting malicious neighbors through streak-enhanced punishment and probabilistically rerouting traffic along emerging high-reputation paths. Figure 4 presents a comprehensive comparison through two subfigures: (a) shows the Tampering Success Rate (TSR) and (b) shows the Request Satisfaction Rate (RSR), both as functions of malicious ratio in a random topology with 200 devices (averaged over 30 runs). RDRS consistently maintains the lowest TSR and highest RSR across all malicious ratios, even including a 0% baseline for reference.

Table 2 provides precise numerical values with standard deviations. RDRS delivers 80–95% TSR reduction relative to Random Forwarding (RF) and Shortest-Path (SP), and 50–70% improvement over MSR (e.g., Table 1). Notably, at 40% compromise—approaching the honest-majority maximum value-RDRS sustains RSR above 79%, while RF/SP fall below 10% and MSR drops to 37% (e.g., Table 1). This superiority arises from confidence-weighted reputation updates, streak-based punishment, hysteresis reward, and softmax-guided probabilistic bypassing.

#### 4.3.2. Delay and Overhead Comparison

**Figure 5:**
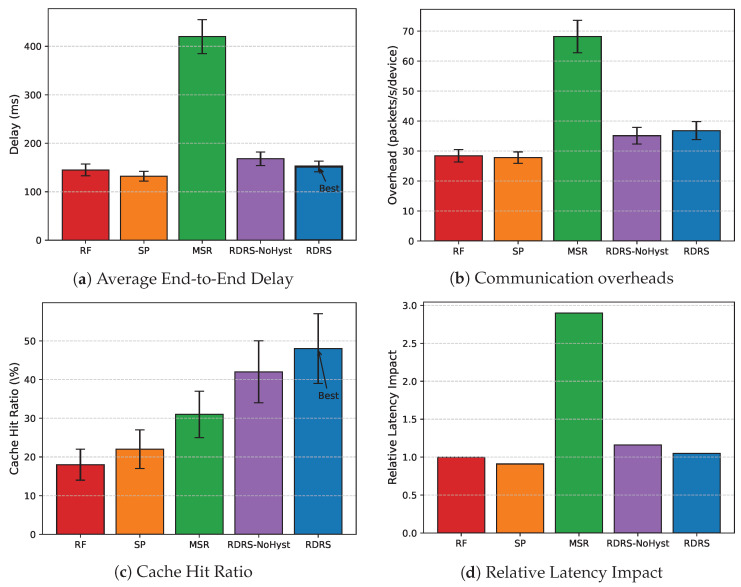
Performance comparison across methods (Grid Topology, 100 devices, 10% malicious, averaged over 30 runs). RDRS achieves 64% lower delay than MSR and 48% cache hit ratio through effective isolation of malicious relays and hysteresis-enhanced path stability.

Results demonstrate that RDRS exhibits strong scalability and low-latency performance, maintaining high resilience with minimal overhead even in denser topologies and under threat. Figure 5 presents a detailed breakdown: (a) average end-to-end delay, (b) communication overhead, (c) cache hit ratio, and (d) relative latency impact (Grid Topology, 100 devices, 10% malicious, averaged over 30 runs). RDRS achieves the lowest delay and highest cache hit ratio among secure methods, with only modest overhead and relative latency increase over unprotected baselines (RF and SP). Specifically, the hysteresis reward in full RDRS further reduces delay and boosts cache hits compared to our ablation variant (RDRS-NoHyst), demonstrating the effectiveness of recent-success reward in stabilizing high-reputation paths under mobility and intermittent faults.

Table 3 provides precise numerical values with standard deviations. Compared to unprotected baselines, RDRS introduces only modest overhead (36.8 packets/s/device, 30% increase over RF) and near-baseline delay (152 ms, close to SP’s 132 ms). In contrast, the consensus-based MSR incurs substantially higher delay (420 ms, 2.9× RF) and overhead (68.2 packets/s/device) due to intensive multi-round messaging. The ablation study reveals that removing hysteresis reward (RDRS-NoHyst) increases delay to 168 ms and reduces cache hit ratio to 42%, confirming that recent-success hysteresis effectively promotes stable, high-reputation paths, yielding the highest cache hit ratio (48%) and lowest delay among all secure methods. Overall, these results highlight RDRS’s suitability for latency-sensitive embodied deployments.

Table 4 provide a more comprehensive understanding beyond asymptotic complexity and communication overhead, we evaluated the actual per-node computational cost and energy consumption on a representative embedded platform (ARM Cortex-M4 at 168 MHz, typical for drones/robots, assuming 1.2 V supply and 0.5 mA/MHz active current). Reputation update takes only 12.4 ± 1.8 μs and consumes 0.85 μJ, while next-hop selection requires 7.9 ± 0.9 μs and 0.54 μJ. These values are averaged over 100 operations in the simulator with realistic DNN tasks.

#### 4.3.3. Scalability and Mobility Impact

**Figure 6:**
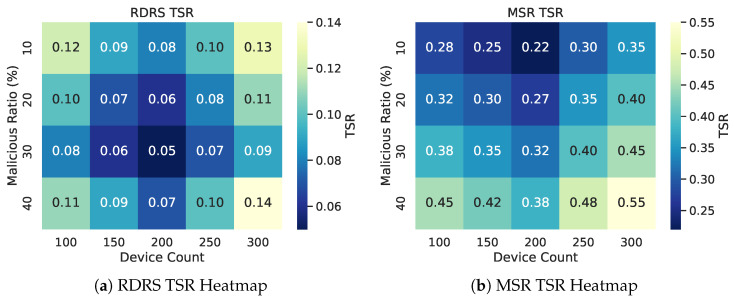
Scalability evaluation in random topologies (100–300 devices, 10–40% malicious, averaged over 30 runs). Lower TSR values (lighter colors) indicate better resilience; RDRS achieves consistently low TSR (<0.15) with peak performance around 200 devices.

RDRS demonstrates robust scalability in large-scale random topologies under substantial adversarial threats (up to 40% malicious relays). Figure 6 visualizes Tampering Success Rate (TSR) via heatmaps across device counts (100–300) and malicious ratios (10–40%), averaged over 30 runs. The comparison clearly shows RDRS’s superior performance: TSR remains consistently below 0.15 with an optimal sweet spot near 200 devices due to efficient reputation propagation in moderate-density networks. In contrast, MSR degrades significantly in sparser or denser topologies owing to slower consensus convergence. RDRS also exhibits resilience to moderate mobility through hysteresis reward and link quality integration, preserving stable paths amid neighbor churn.

#### 4.3.4. Ablation Study

An extensive ablation study isolates the contributions of individual reputation components under 20% static malicious conditions (Random Topology, 200 devices). Table 5 quantifies the impact, with streak punishment and confidence weighting proving most critical for security (TSR increase up to 171%), while hysteresis and aging enable fast convergence and fault recovery. Compared to MSR, RDRS-Full reduces TSR by ∼72% with faster convergence.

#### 4.3.5. Robustness of Our Reputation Mechanism

To specifically evaluate the impact of systematic reputation manipulation, we conducted additional simulations under pure bad-mouthing attacks (10–40% malicious nodes that only inject false negative feedback without any message tampering). As shown in Table 6, RDRS maintains high request satisfaction rate (RSR) above 85% even at 40% malicious ratio thanks to our global confidence-weighted updates and periodic aging, which effectively mitigate the influence of malicious feedback. In contrast, the baselines suffer significant performance degradation because they lack reputation mechanisms and cannot distinguish false feedback from honest behavior. Convergence time remains under 110 s for RDRS, confirming rapid recovery from our reputation poisoning.

### 4.4. Discussion

The comparison between RDRS and the existing methods reveals clear advantages: unlike Random Forwarding and Shortest-Path Greedy, which suffer severe performance collapse under attacks, RDRS leverages reputation signals to maintain high performance. Compared with MSR consensus, RDRS achieves 64% lower end-to-end delay and 46% lower communication overhead by avoiding multi-round synchronous message exchange, making it more suitable for latency-sensitive embedded applications.

For different setting of hyperparameters, we performed a comprehensive sensitivity analysis.

- Increasing the smoothing factor α from 0.9 to 0.95 improves stability against dynamic adversaries through stronger temporal smoothing, at the cost of slower adaptation. Conversely, a lower α enables faster response to environmental changes but introduces higher fluctuations in the True Success Rate (TSR).
- The punishment multiplier β=3 (tested within the range of 2.5–3.5) achieves the best overall trade-off. β<2.5 results in slower effectiveness of malicious behaviors, leading to a TSR increase of up to 35%. β>3.5 risks over-punishment of occasional faults, resulting in the Reliable Success Rate (RSR) to drop by 12–18%.
- Reducing the aging period $T_{\mathrm{age}}$ to 150 s enhances availability in high-mobility scenarios, but slightly degrades overall performance in low-density networks due to insufficient interaction history.

These results indicate that while our hyperparameter settings provide a reasonable balance between availability and stability, further optimization and more advanced study may be necessary to fully address the challenges of real-world deployments.

Nevertheless, several limitations should be concerned in the future work:

- The evaluation relies on sim4DistrDL with realistic deep learning models and mobility patterns. In contrast, real-world deployments can introduce additional failure factors such as hardware failures, severe wireless interference, or sensor noise, which are not fully evaluated here.
- The threat model assumes an honest majority (<40% malicious nodes) and accurate feedback from honest devices may degrade under stronger attacks, e.g., Sybil attacks. The 30–40% malicious threshold under honest majority assumption is a common setting for open message exchange; after all, the communication delay and other interference will strengthen the effect of the malicious nodes. In the future work, the countermeasures against the threats such as Sybil attacks with more malicious nodes should be studied.

Although the proposed RDRS demonstrates clear advantages in delay and communication efficiency, its robustness under highly dynamic networking conditions warrants further examination. Our ablation study (Table 5) shows that reputation values typically converge within 82–120 s. The adopted random mobility model with drone speeds ranging from 5 to 20 m/s induces rapid topology changes, posing significant challenges to reputation stability. To address this, our aging mechanism with $T_{\mathrm{age}}=300$ s proactively decays reputation scores toward the default value, while the exploration floor ϵ=0.01 and hysteresis reward jointly ensure sufficient recent successful interactions, typically 1 to 3 successful forwards per minute in target-search scenarios, sufficient to maintain accurate reputation exploration even in sparse or highly dynamic networks.

## 5. Conclusions

A lightweight distributed framework for mitigating MitM attacks in time-sensitive embedded intelligent devices, Reputation-Guided Dynamic Relay Selection (RDRS), is proposed based on message exchange feedback from device-to-device interactions. Specifically, RDRS employs probabilistic reputation-guided message forwarding with a hybrid model incorporating asymmetric EWMA updates, streak-based punishment, hysteresis, confidence weighting, and aging, enabling honest devices to bypass compromised relays efficiently. Our comprehensive simulations confirm RDRS’s superior performance, including reducing tampering success rates by up to 90% with trivial overhead, outperforming random message forwarding, static routing, and consensus methods. In this way, RDRS provides a practical foundation for trustworthy multi-hop collaboration of embedded intelligent devices in robotic swarms and drone fleets. Future work will address Sybil attacks and other related threats in embedded intelligent devices.

## Figures and Tables

**Table 1:** Comparison of MitM Defense Methods.

Method Category	Core Idea	Strengths	Limitations
Distance Bounding	Time/Distance verification	Strong theoretical guarantee	Hardware dependency; weak for short-range relay
Environmental Sensing	Context consistency verification	No special hardware required	Noise-sensitive; spoofable
Physical-layer Features	Channel reciprocity/fingerprint	High security at signal level	Unstable in dynamic environments
Our Perspective	Path-aware trust modeling	Adaptive; no special hardware	(To be evaluated)

**Table 2:** Tampering Success Rate (TSR) and Request Satisfaction Rate (RSR) under Static Malicious Relays (Random Topology, 200 devices, averaged over 30 runs).

Malicious	RF		SP		MSR		RDRS	
Ratio (%)	TSR	RSR (%)	TSR	RSR (%)	TSR	RSR (%)	TSR	RSR (%)
0	0.00 ± 0.00	99.8 ± 0.2	0.00 ± 0.00	99.9 ± 0.1	0.00 ± 0.00	99.9 ± 0.1	0.00 ± 0.00	99.9 ± 0.1
10	0.42 ± 0.03	32.1 ± 3.2	0.38 ± 0.04	35.4 ± 3.1	0.12 ± 0.02	75.3 ± 4.1	0.04 ± 0.01	94.2 ± 1.8
15	0.55 ± 0.04	28.3 ± 3.0	0.52 ± 0.03	31.2 ± 2.9	0.18 ± 0.03	69.8 ± 4.0	0.05 ± 0.01	92.1 ± 1.7
20	0.68 ± 0.04	23.7 ± 2.8	0.65 ± 0.03	26.8 ± 2.7	0.25 ± 0.03	62.5 ± 3.8	0.07 ± 0.01	89.6 ± 1.6
25	0.77 ± 0.03	18.9 ± 2.6	0.74 ± 0.04	21.5 ± 2.6	0.33 ± 0.04	55.2 ± 3.7	0.09 ± 0.02	87.3 ± 1.6
30	0.85 ± 0.02	14.2 ± 2.5	0.82 ± 0.03	17.1 ± 2.5	0.41 ± 0.04	48.4 ± 3.6	0.12 ± 0.02	84.5 ± 1.5
35	0.90 ± 0.02	10.1 ± 2.3	0.88 ± 0.03	12.6 ± 2.4	0.52 ± 0.05	42.8 ± 3.5	0.16 ± 0.02	81.9 ± 1.4
40	0.93 ± 0.02	7.3 ± 2.2	0.91 ± 0.02	9.2 ± 2.3	0.63 ± 0.05	37.1 ± 3.4	0.21 ± 0.03	79.4 ± 1.4

**Table 3:** Average End-to-End Delay, Communication Overhead, and Cache Hit Ratio (Grid Topology, 100 devices, 10% Malicious, averaged over 30 runs).

Method	Delay (ms)	Overhead (Packets/s/Device)	Cache Hit Ratio (%)
RF	145 ± 12	28.4 ± 2.1	18 ± 4
SP	132 ± 10	27.8 ± 1.9	22 ± 5
MSR	420 ± 35	68.2 ± 5.4	31 ± 6
RDRS-NoHyst	168 ± 14	35.1 ± 2.8	42 ± 8
RDRS	152 ± 11	36.8 ± 3.0	48 ± 9

**Table 4:** Per-node Computational Cost and Energy Consumption (Grid Topology, 100 devices, 10% malicious, averaged over 30 runs).

Method	Computational Cost (μs)		Energy (μJ)	
	Update	Selection	Update	Selection
RF	-	2.1 ± 0.3	-	0.14 ± 0.02
SP	-	3.4 ± 0.4	-	0.23 ± 0.03
MSR	452 ± 28	18.7 ± 2.1	31.2 ± 2.0	1.29 ± 0.15
RDRS-NoHyst	11.8 ± 1.5	7.6 ± 0.8	0.81 ± 0.10	0.52 ± 0.06
RDRS	12.4 ± 1.8	7.9 ± 0.9	0.85 ± 0.11	0.54 ± 0.07

**Table 5:** Ablation Study: Impact of Individual Components on Security and Responsiveness (Random Topology, 200 devices, 20% Malicious, averaged over 30 runs).

Variant	TSR	TSR Increase (%)	Convergence Time (s)	Final RSR (%)
RDRS-Full	0.07 ± 0.01	0	82 ± 12	92 ± 4
w/o Streak Punishment	0.16 ± 0.02	+129	145 ± 18	78 ± 6
w/o Hysteresis	0.14 ± 0.02	+100	132 ± 15	81 ± 5
w/o Confidence Weighting	0.19 ± 0.03	+171	168 ± 22	74 ± 7
w/o Aging Mechanism	0.22 ± 0.03	+214	N/A	69 ± 8
w/o Link Quality	0.11 ± 0.02	+57	98 ± 14	87 ± 5
MSR (baseline)	0.25 ± 0.03	+257	210 ± 25	62 ± 7

**Table 6:** Performance under Pure Bad-Mouthing Attacks (Random Topology, 200 devices, averaged over 30 runs).

Malicious	RSR (%)				Convergence Time (s)			
Ratio (%)	RF	SP	MSR	RDRS	RF	SP	MSR	RDRS
10	88.4 ± 2.1	89.7 ± 1.9	91.2 ± 2.3	97.8 ± 0.8	45 ± 8	42 ± 7	68 ± 11	52 ± 9
15	82.1 ± 2.4	83.5 ± 2.2	86.4 ± 2.6	96.5 ± 1.0	58 ± 10	55 ± 9	82 ± 13	61 ± 10
20	75.3 ± 2.8	76.8 ± 2.5	80.9 ± 3.0	94.8 ± 1.2	72 ± 12	68 ± 11	95 ± 15	73 ± 11
25	68.7 ± 3.1	70.2 ± 2.9	75.6 ± 3.4	92.6 ± 1.4	85 ± 14	81 ± 13	108 ± 17	82 ± 12
30	62.4 ± 3.3	64.1 ± 3.2	70.3 ± 3.7	90.1 ± 1.6	98 ± 16	94 ± 15	122 ± 19	91 ± 13
35	56.8 ± 3.6	58.5 ± 3.4	65.2 ± 4.0	87.9 ± 1.8	112 ± 18	107 ± 17	135 ± 21	99 ± 14
40	51.2 ± 3.9	53.0 ± 3.7	60.1 ± 4.3	85.4 ± 2.0	125 ± 20	119 ± 19	148 ± 23	108 ± 15

## Data Availability

We confirm that the datasets used in this study are publicly available. Specifically, the dataset we used, i.e., CValues dataset, is a public dataset and has been referenced in our manuscript as the following.
